# Establishment of a rat model of lumbar facet joint osteoarthritis using intraarticular injection of urinary plasminogen activator

**DOI:** 10.1038/srep09828

**Published:** 2015-04-20

**Authors:** Feng Shuang, Shu-Xun Hou, Jia-Liang Zhu, Yan Liu, Ying Zhou, Chun-Li Zhang, Jia-Guang Tang

**Affiliations:** 1 Department of Orthopaedics, The First Affiliated Hospital of General Hospital of Chinese PLA, Beijing 100048, China; 2 Department of Orthopedics, The 94th Hospital of Chinese PLA, Nanchang 330002, China

## Abstract

Lumbar facet joint (LFJ) osteoarthritis (OA) is an important etiology of low back pain. Several animal models of LFJ OA have been established using intraarticular injection of various chemicals. This study aimed to establish a rat model of LFJ OA using urinary plasminogen activator (uPA). Sprague-Dawley rats were treated with intraarticular injection in the L5-L6 facet joints with uPA (OA group, n = 40) or normal saline (vehicle group, n = 40). Mechanical and thermal hyperalgesia in the ipsilateral hind paws were evaluated using von Frey hairs and a thermoalgesia instrument, respectively. Toluidine blue staining, hematoxylin-eosin staining, and immunohistochemical examination of the LFJ was performed. Treatment with uPA induced cartilage damage, synovitis, and proliferation of synovial cells in the fact joints. The OA group showed significantly higher hyperalgesia in the hind paws in comparison with the vehicle group and normal controls (*P* < 0.05). Expression of IL-1β, TNF-α, and iNOS in the LFJ cartilage in the OA group was significantly increased (P < 0.05). A rat model of LFJ OA was successfully established using intraarticular injection of uPA. This animal model is convenient and shows good resemblance to human OA pathology.

Lumbar facet joints (LFJ), also known as the zygapophyseal joints, together with the intervertebral disc, constitute the so-called “three-joint complex” in the spine. LFJ in the posterior aspect of the vertebral column are the only true synovial joints between adjacent spinal levels in humans. Injury and stress overload can result in osteoarthritis (OA) in the LFJ as well as adjacent parts of the lumbar spine. LFJ OA has been shown to be an important source of low back pain^1^. Low back pain is a prevalent problem affecting up to 80% of the population, leading to limited activity and decreased quality of life^2^,^3^,^4^. However, the specific mechanisms of LFJ OA in the development of low back pain are still not clear.

Animal models are useful tools for the investigation of association between animal behavioral and LFJ OA. Intervertebral disc degeneration and LFJ OA in an animal model using intraarticular injection of collagenase has been reported^5^. Similar to collagenase, urinary plasminogen activator (uPA) can cause lysis of extracellular matrix and basement membrane, leading to inflammatory joint diseases^6^,^7^. In this study, we established a rat model of LFJ OA using intraarticular injection of uPA and evaluated the various animal behavior and pathological changes.

## Methods

### Ethical statement

All experimental protocol were approved by the Ethical Committee of The First Affiliated Hospital of Chinese PLA General Hospital. The methods were carried out in accordence with the approved guidelines.

### Animals

Male Sprague-Dawley rats weighing 250–300 g (n = 88) were provided by the Laboratory Animal Center of Academy of Military Medical Sciences. The procedures used in this study were in agreement with the guidelines of laboratory animal care^8^. The animals were kept at room temperature of 22–26°C with free access to food and water and 12-h light/dark cycle.

### Induction of OA

The rats were anesthetized with intraperitoneal injection of 1% pentobarbital sodium at a dose of 40 mg/kg. The rats were then put into a prone position and a 1.5-cm posterior medial incision was carried out. The left paraspinal muscles were retracted to expose the left L5-L6 facet joint. A 34G needle (WPI, US) was attached to a 5-μL microsyringe (WPI, US) and inserted into the articular cavity. The OA group (n = 40) was treated with intraarticular injection of 2 mg/L uPA in 5 μL normal saline. The vehicle group (n = 40) was treated with 5 μL normal saline. After injection, the needle was kept in place for 15 s before withdrawal. Then the wound was sutured. Eight rats were not specially treated and served as normal controls.

### Test of mechanical hyperalgesia

The animals were tested for mechanical hyperalgesia using the von Frey hairs (North Coast Medical USA) before injection and on postoperative days 3, 7, 14, 28, 42, and 56 (n = 8). Animals were habituated to the wire mesh bottom cages for 30 min each day for three days before the test. A serious of von Frey hairs in ascending order of force (1.0 g, 1.4 g, 2.0 g, 4.0 g, 6.0 g, 8.0 g, 10.0 g, 15.0 g, and 26 g) were applied to the plantar surface of the ipsilateral hind paws for 6 s. If withdrawal response was not induced, the next level of von Frey hair was applied until at least 5 withdrawal occurred in 10 stimuli (50% positive). The minimal force required to elicit 50% positive response was recorded as the paw withdrawal threshold.

### Test of thermal hyperalgesia

To evaluate the thermal hyperalgesia, the thermal withdrawal latency of the ipsilateral hind paw was tested using a thermoalgesia instrument (BW-PL-200, Ruanlong, Shanghai) before injection and on postoperative days 3, 7, 14, 28, 42, and 56 (n = 8)^9^. The rats were placed on a glass plate of constant temperature for 15 min before each test. The ipsilateral hind paws were irradiated by the thermoalgesia instrument from below the glass plate. The time (s) from irradiation to paw withdrawal was recorded as the thermal withdrawal latency. Each test was repeated three times with 10 min interval. To avoid skin burn, the maximum irradiation time was set as 60 s.

### Gait analysis

Gait analysis was performed using a CatWalk system (XT10.5, Noldus, Netherland) before injection and on postoperative days 3, 7, 14, 28, 42, and 56 (n = 8) as previously described^10^. Briefly, the animals were placed on a glass plate and allowed to walk through. Light was emitted from a LED and completely reflected internally in the glass plate. Contacts between the rat's paw and the glass surface would allow light to reflect downwards, showing a sharp image of the paw print. A high-speed camera below the glass plate recorded the footprint of the rats and send the images to the CatWalk system for analysis. The system analyzed the following parameters:

- Print area (cm^2^), the area of contact between the plantar surface of the ipsilateral hind paws and the glass plate;
- Stand phase (s), the duration of contact between the paw and the glass plate in one step cycle, reflecting the limb pain intensity;
- Swing phase (s), the duration of non-contact with the glass plate in one step cycle, also reflecting the limb pain intensity;
- Swing speed (cm/s), the speed of the paw movement in one step cycle, which was calculated by dividing the one step length by the swing phase.

### Toluidine blue staining

On postoperative days 7, 14, 28, 42, and 56, eight rats were randomly chosen from the OA group and the vehicle group and euthanized by intraperitoneal overdose injection of pentobarbital sodium. The normal control group was sacrificed on postoperative day 56. The L5-L6 spine was removed en bloc, fixed in 4% paraformaldehyde for 48 hours, decalcified, and embedded in paraffin. The block was cut into 4-μm sections on the coronal plane and stained with toluidine blue. The sections were scored using the Osteoarthritis Research Society International (OARSI) histopathology grading system^11^,^12^. In this system, the grade of damage from 0 to 6 is defined as the depth of progression of OA into the cartilage and the stage of damage is defined as the horizontal extent of cartilage involvement from 0 to 4. The final score is the combined value of grade and stage (score range 0–24).

### Hematoxylin-eosin (HE) staining of the synovium

To evaluate the condition of synovitis, HE staining was performed using the sections of synovium of the L5-L6 facet joints. Synovitis was scored with a 9-point scale including three criteria. The proliferation of synovial cells was scored as follows: fewer than three layers (0), three to four layers (1), five to six layers (2), and more than six layers (3). Inflammation was scored as follows: no lymphocytes infiltration (0), lymphocytes aggregation (1), lymphoid follicles (2), and lymphoid follicles with germinal center formation (3). Angiogenesis was scored as follows: none (0), mild (1), moderate (2), and severe (3).

### Immunohistochemical staining

The sections were incubated with various rabbit-anti rat monoclonal antibodies (IL-1β, 1:50; TNF-α, 1:50; iNOS, 1:200; Abcam, US) at 4°C overnight. Biotin-labeled goat-anti rabbit secondary antibody was added for 1 hour at room temperature. The staining was displayed using Diaminobenzidine. The positivity cells were stained with brown color. The expression of the targeted molecules were calculated by multiplying the staining intensity score with the positive cell score. The staining intensity was scored as the following: no staining (0), light staining (1), moderate staining (2), and strong staining (3). The positive cells in three randomly selected field were scored as the following: <10% positive cells (0), 11%–50% positive cells (2), 51%–75% positive cells (3), and >75% (4).

### Statistical analysis

Continuous data were expressed as mean ± standard deviation (SD). The data were tested to affirm normal distribution. Comparison was made using one-way ANOVA with SPSS 17.0 software (SPSS, US). *P* < 0.05 was regarded as statistically significant.

## Results

### Paw withdrawal threshold

On postoperative days 3, 7, and 14, the OA group showed significantly lower paw withdrawal threshold in comparison with the vehicle group and the normal control group (*P* < 0.05, Table 1), suggesting significantly increased mechanical hyperalgesia in the rats with induced LFJ OA.

### Thermal withdrawal latency

On postoperative days 3, 7, and 14, the OA group showed significantly shorter thermal withdrawal latency in comparison with the vehicle group and the normal control group (*P* < 0.05, Table 2), suggesting significantly increased thermal hyperalgesia in the rats with induced LFJ OA.

### Gait analysis

On postoperative days 3, 7, and 14, the OA group showed significantly lower print area, stand phase, swing speed, and significantly higher swing phase in comparison with the vehicle group and the normal control group (*P* < 0.05, Tables 3–6), suggesting significantly increased limb pay intensity in the rats with induced LFJ OA.

### Histopathologic analysis

**Figure 1:**
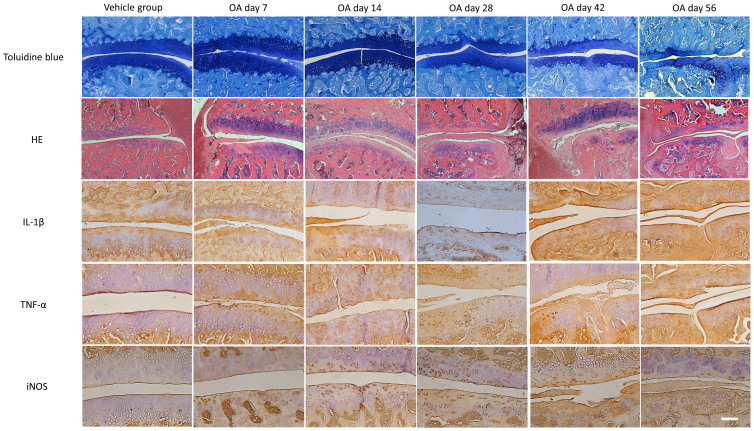
Toluidine blue staining, HE staining, and immunohistochemical staining of the LFJ. Scale bar: 100 μm.

The toluidine blue staining, HE staining, and immunohistochemical staining results are shown in Fig. 1. Toluidine blue staining showed severe cartilage damage, cartilage erosion, and joint space narrow in the LFJ injected with uPA. HE staining showed proliferation of the synovial cells, lymphocyte infiltration, and degenerative changes in the cartilage in the OA group. The OA group showed significantly higher OARSI scores and synovitis scores than the vehicle group (P < 0.05, Table 7 and 8). The expressions of IL-1β, TNF-α, and iNOS in the cartilage were significantly increased in the OA group in comparison with the vehicle group (*P* < 0.05).

## Discussion

LFJ OA has been implied to be a major contributing factor for low back pain. There has been previously reported rat models of LFJ OA established using intraarticular injection of collagenase^5^, monosodium iodoacetate^13^,^14^, and complete Freund's adjuvant^15^. In our study, we tried to establish a rat model of LFJ OA using intraarticular injection of uPA. uPA is a serine protease and its primary physiological substrate is plasminogen. Plasminogen is an inactive form (zymogen) of the serine protease plasmin. Activation of plasmin triggers a proteolysis cascade that, depending on the physiological environment, participates in thrombolysis or extracellular matrix degradation^16^,^17^. It has been found that uPA plays important roles in the degeneration of extracellular matrix in the osteoarthritic cartilage^6^, and levels of uPA in the articular cartilage and synovium are associated with the severity of cartilage damage^18^. Regarding the pathological roles of uPA in OA, we speculate that uPA might be a good candidate for the establishment of animal models of LFJ OA. In our study, we injected uPA into the articular space of L5-L6 facet joint in rats. Histopathologic examination showed that uPA induced synovitis and cartilage degeneration in the LFJ, which mimics the pathological changes in human OA. In comparison with Freund's adjuvant, uPA was milder and this uPA-induced model can reflect the complete pathological process of LFJ OA of its early, middle, and late phases. Therefore, the uPA-induced model might be more useful for the development of therapeutic methods targeting the early and middle phases of LFJ OA.

Mechanical hyperalgesia was found in a rat model of LFJ OA induced by intraarticular injection of complete Freund's adjuvant^15^. In addition, concomitant pain-related behavioral changes were also noticed. It is suggested that facet joint inflammation, and not degeneration of the joint, might have been a cause of the hyperalgesia. In our study, both mechanical and thermal hyperalgesia were tested to gain further insight into this issue. We found that intraarticular injection of uPA into the LFJ in rats significantly decreased the paw withdrawal threshold and thermal withdrawal latency, indicating the presence of mechanical and thermal hyperalgesia. Gait analysis also found that the animal behaviors showed significant changes in the OA group. The specific mechanisms underlying the hyperalgesia and gait changes are not clear. Our results indicate that some inflammatory factories might play a role. IL-1β is involved in the development neuropathic pain, and can also increase the sensitivity of the spine to pain stimuli^19^. The expression of TNF-α and iNOS in the LFJ cartilage in rats with induced LFJ OA was also increased. IL-1β and TNF-α are scarcely expressed in normal facet joints and are associated with the inflammation severity in LFJ OA. iNOS can induce the synthesis of NO in OA conditions, and NO is involved in the apoptosis of chondrocytes^20^. Chondrocytes apoptosis can lead to cartilage damage and destabilized facet joint structure.

In conclusion, we successfully established a rat model of LFJ OA using intraarticular injection of uPA. This animal model is convenient and shows good resemblance of human facet joint OA pathology. Further investigation is needed to find out the mechanisms of hyperalgesia in this animal model.

## Author Contributions

**Author contribution** F.S. and S.X.H. wrote the main manuscript text, J.L.Z., Y.L. and Y.Z. collected data and carried out the experiments, C.L.Z. analyzed the data, J.G.T. designed the experiments. All author has approved the manuscript.

## Figures and Tables

**Table 1:** Comparison of paw withdrawal threshold among the OA group, vehicle group, and normal control group (n = 8, mean ± SD, g)

Groups	Before injection	Day 3	Day 7	Day 14	Day 28	Day 42	Day 56
OA	17.75 ± 5.09	7.25 ± 1.83*	7.75 ± 1.69*	5.75 ± 1.67*	10.50 ± 2.93	13.13 ± 2.59	13.75 ± 2.32
Vehicle	19.13 ± 5.69	11.63 ± 2.88	16.88 ± 6.13	16.38 ± 3.89	15.13 ± 4.94	17.13 ± 5.74	17.75 ± 5.09
Normal control	18.50 ± 6.44	20.5 ± 5.88	18.63 ± 6.99	17.13 ± 5.74	19.13 ± 5.69	19.88 ± 6.75	17.88 ± 7.04

*, vs the vehicle group and normal control group, *P* < 0.05

**Table 2:** Comparison of thermal withdrawal latency among the OA group, vehicle group, and normal control group (n = 8, mean ± SD, s)

Groups	Before injection	Day 3	Day 7	Day 14	Day 28	Day 42	Day 56
OA	41.23 ± 5.18	22.25 ± 2.89*	24.82 ± 3.45*	21.94 ± 3.25*	35.28 ± 3.21*	38.78 ± 4.06	39.08 ± 3.87
Vehicle	42.45 ± 5.41	36.28 ± 4.05	39.31 ± 4.37	40.49 ± 3.32	42.06 ± 3.55	40.53 ± 4.14	41.17 ± 3.96
Normal control	40.87 ± 6.25	39.29 ± 5.17	41.56 ± 5.34	43.86 ± 4.98	43.87 ± 5.56	42.63 ± 5.79	42.57 ± 5.67

*, vs the vehicle group and normal control group, *P* < 0.05

**Table 3:** Comparison of print area among the OA group, vehicle group, and normal control group (n = 8, mean ± SD, cm^2^)

Groups	Before injection	Day 3	Day 7	Day 14	Day 28	Day 42	Day 56
OA	1.214 ± 0.064	0.998 ± 0.061*	1.114 ± 0.072*	0.893 ± 0.048*	1.167 ± 0.057	1.175 ± 0.063	1.188 ± 0.059
Vehicle	1.227 ± 0.075	1.124 ± 0.058	1.197 ± 0.063	1.213 ± 0.076	1.207 ± 0.069	1.223 ± 0.077	1.219 ± 0.071
Normal control	1.195 ± 0.069	1.231 ± 0.068	1.244 ± 0.076	1.198 ± 0.074	1.206 ± 0.072	1.219 ± 0.068	1.235 ± 0.078

*, vs the vehicle group and normal control group, *P* < 0.05

**Table 4:** Comparison of stand phase among the OA group, vehicle group, and normal control group (n = 8, mean ± SD, s)

Groups	Before injection	Day 3	Day 7	Day 14	Day 28	Day 42	Day 56
OA	0.332 ± 0.037	0.275 ± 0.013*	0.257 ± 0.015*	0.238 ± 0.017*	0.286 ± 0.021*	0.296 ± 0.018	0.308 ± 0.026
Vehicle	0.325 ± 0.036	0.297 ± 0.018	0.307 ± 0.027	0.311 ± 0.028	0.316 ± 0.026	0.321 ± 0.037	0.318 ± 0.032
Normal control	0.317 ± 0.033	0.312 ± 0.024	0.321 ± 0c.034	0.316 ± 0.028	0.333 ± 0.035	0.335 ± 0.042	0.326 ± 0.027

*, vs the vehicle group and normal control group, *P* < 0.05

**Table 5:** Comparison of swing phase among the OA group, vehicle group, and normal control group (n = 8, mean ± SD, s)

Groups	Before injection	Day 3	Day 7	Day 14	Day 28	Day 42	Day 56
OA	0.129 ± 0.008	0.163 ± 0.013*	0.161 ± 0.012*	0.183 ± 0.015*	0.167 ± 0.017*	0.153 ± 0.018	0.146 ± 0.016
Vehicle	0.132 ± 0.011	0.147 ± 0.012	0.144 ± 0.011	0.138 ± 0.013	0.134 ± 0.009	0.137 ± 0.008	0.138 ± 0.015
Normal control	0.136 ± 0.012	0.133 ± 0.010	0.142 ± 0.009	0.135 ± 0.009	0.128 ± 0.010	0.140 ± 0.013	0.137 ± 0.011

*, vs the vehicle group and normal control group, *P* < 0.05

**Table 6:** Comparison of swing speed among the OA group, vehicle group, and normal control group (n = 8, mean ± SD, cm/s)

Groups	Before injection	Day 3	Day 7	Day 14	Day 28	Day 42	Day 56
OA	107.32 ± 5.76	84.64 ± 4.26*	80.31 ± 6.43*	77.85 ± 5.52*	92.54 ± 7.58*	97.32 ± 8.81*	99.69 ± 9.12
Vehicle	110.64 ± 6.21	99.78 ± 7.73	104.56 ± 8.94	107.84 ± 8.51	107.89 ± 9.23	108.57 ± 8.47	104.85 ± 6.22
Normal control	106.87 ± 6.38	109.52 ± 8.43	112.13 ± 9.65	105.95 ± 9.78	103.56 ± 8.48	111.21 ± 10.14	108.73 ± 8.83

*, vs the vehicle group and normal control group, *P* < 0.05

**Table 7:** Comparison of OARSI scores between the OA group and the vehicle group (n = 8, mean ± SD)

Groups	Day 7	Day 14	Day 28	Day 42	Day 56
OA	1.75 ± 0.46*	5.75 ± 1.67*	8.13 ± 0.99*	12.50 ± 2.33*	17.50 ± 1.69*
Vehicle	0	0	0	0.25 ± 0.46	0.50 ± 0.54

*, vs the vehicle group, *P* < 0.05

**Table 8:** Comparison of synovitis scores between the OA group and the vehicle group (n = 8, mean ± SD)

Groups	Day 7	Day 14	Day 28	Day 42	Day 56
OA	2.75 ± 0.71*	3.88 ± 0.64*	6.75 ± 0.71*	5.00 ± 0.76*	4.13 ± 0.84*
Vehicle	0.25 ± 0.46	0.50 ± 0.54	0.38 ± 0.54	0.63 ± 0.52	0.50 ± 0.54

*, vs the vehicle group, *P* < 0.05
